# Structural basis of an epitope tagging system derived from *Haloarcula marismortui* bacteriorhodopsin I D94N and its monoclonal antibody GD‐26

**DOI:** 10.1111/febs.16184

**Published:** 2021-10-01

**Authors:** Po‐Jung Pao, Min‐Feng Hsu, Ming‐Hui Chiang, Chun‐Ting Chen, Cheng‐Chung Lee, Andrew H.‐J. Wang

**Affiliations:** ^1^ Institute of Biological Chemistry Academia Sinica Taipei Taiwan

**Keywords:** 3_10_ helix, bacteriorhodopsin, monoclonal antibody, peptide tag, peptide–antibody complex

## Abstract

Specific antibody interactions with short peptides have made epitope tagging systems a vital tool employed in virtually all fields of biological research. Here, we present a novel epitope tagging system comprised of a monoclonal antibody named GD‐26, which recognises the TD peptide (GTGATPADD) derived from *Haloarcula marismortui* bacteriorhodopsin I (*Hm*BRI) D94N mutant. The crystal structure of the antigen‐binding fragment (Fab) of GD‐26 complexed with the TD peptide was determined to a resolution of 1.45 Å. The TD peptide was found to adopt a 3_10_ helix conformation within the binding cleft, providing a characteristic peptide structure for recognition by GD‐26 Fab. Based on the structure information, polar and nonpolar forces collectively contribute to the strong binding. Attempts to engineer the TD peptide show that the proline residue is crucial for the formation of the 3_10_ helix in order to fit into the binding cleft. Isothermal calorimetry (ITC) reported a dissociation constant *K*_D_ of 12 ± 2.8 nm, indicating a strong interaction between the TD peptide and GD‐26 Fab. High specificity of GD‐26 IgG to the TD peptide was demonstrated by western blotting, ELISA and immunofluorescence as only TD‐tagged proteins were detected, suggesting the effectiveness of the GD‐26/TD peptide tagging system. In addition to already‐existing epitope tags such as the FLAG tag and the ALFA tag adopting either extended or α‐helix conformations, the unique 3_10_ helix conformation of the TD peptide together with the corresponding monoclonal antibody GD‐26 offers a novel tagging option for research.

## Graphical abstract

Specific antibody interactions with short peptides have made epitope tagging systems an indispensable tool in scientific research. We introduced a novel TD epitope tag (GTGATPADD) recognised by a highly specific monoclonal antibody GD‐26. The crystal structure revealed that the TD peptide adopts a 3_10_ helix conformation binding to GD‐26 antigen‐binding fragment. The TD tag has been successfully applied to western blotting, ELISA and immunofluorescence with the use of GD‐26 IgG.

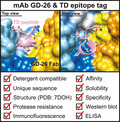

**Abbreviations**
CDR: complementarity‐determining region
eGFP: enhanced GFP
Fab: antigen‐binding fragment
*Hm*BRI: *Haloarcula marismortui* bacteriorhodopsin I
ITC: isothermal titration calorimetry
V_H_
: heavy‐chain variable domain
V_L_
: light‐chain variable domain

## Introduction

Epitope tags are short peptide sequences (usually 6–15 amino acids in length) that are fused to a target protein for specific anti‐tag antibody/tag recognition. Since the introduction of an epitope tag for recombinant protein detection [1], epitope tagging systems have become an indispensable tool in many fields of scientific research. Initially, they were developed to facilitate recombinant protein purification and detection. Epitope tagging systems are of great advantage, especially when antibodies to the protein of interest are unavailable. Epitope tags are described as a single tool that can purify numerous proteins. Later, the use of epitope tags has been explored and applied to a wide range of biological applications including immunoprecipitation, immunofluorescence microscopy [2], protein trafficking in cells [3, 4], protein crystallisation and structural biology [5].

Antibodies for the most widely used epitope tagging systems such as the FLAG tag [6], the HA tag [7, 8], the c‐Myc tag [9, 10] and the polyhistidine tag [11] are all made commercially available. However, each tag has its own strengths and weaknesses and researchers need to choose the most suitable tagging systems for their experiments [12, 13]. For instance, the polyhistidine tag is convenient for protein purification with a mild elution condition, but it lacks high specificity leading to poor results in immunoblotting. On the other hand, although the FLAG tag system is not as convenient as the polyhistidine tag for the purpose of protein purification, it is highly specific and often used for immunoblotting. It is therefore common to include a combination of multiple tagging systems in one expression construct in order to fulfil various needs [14, 15].

Epitope tags are short enough that they are less likely to interfere with the structure and the activity of a target protein. However, the presence of epitope tags may still affect the structure [16, 17], stability [18] and function [19] of the protein of interest [13]. Besides, the FLAG tag has been found to undergo a post‐translational modification, which disrupts the anti‐tag antibody/tag interactions resulting in a decrease in the purification yield [20]. Despite the small size and presumed inertness of epitope tags, the potential negative effects of epitope tags on the protein of interest need to be taken into account. Studying the molecular interaction of an anti‐tag antibody/tag complex at the atomic level by X‐ray crystallography helps understand, predict and improve the performance of an epitope tag. The development of new epitope tags and epitope engineering remains as an ongoing process in order to offer a greater range of epitope tags with distinct characteristics for researchers to choose from.

**Fig. 1:**
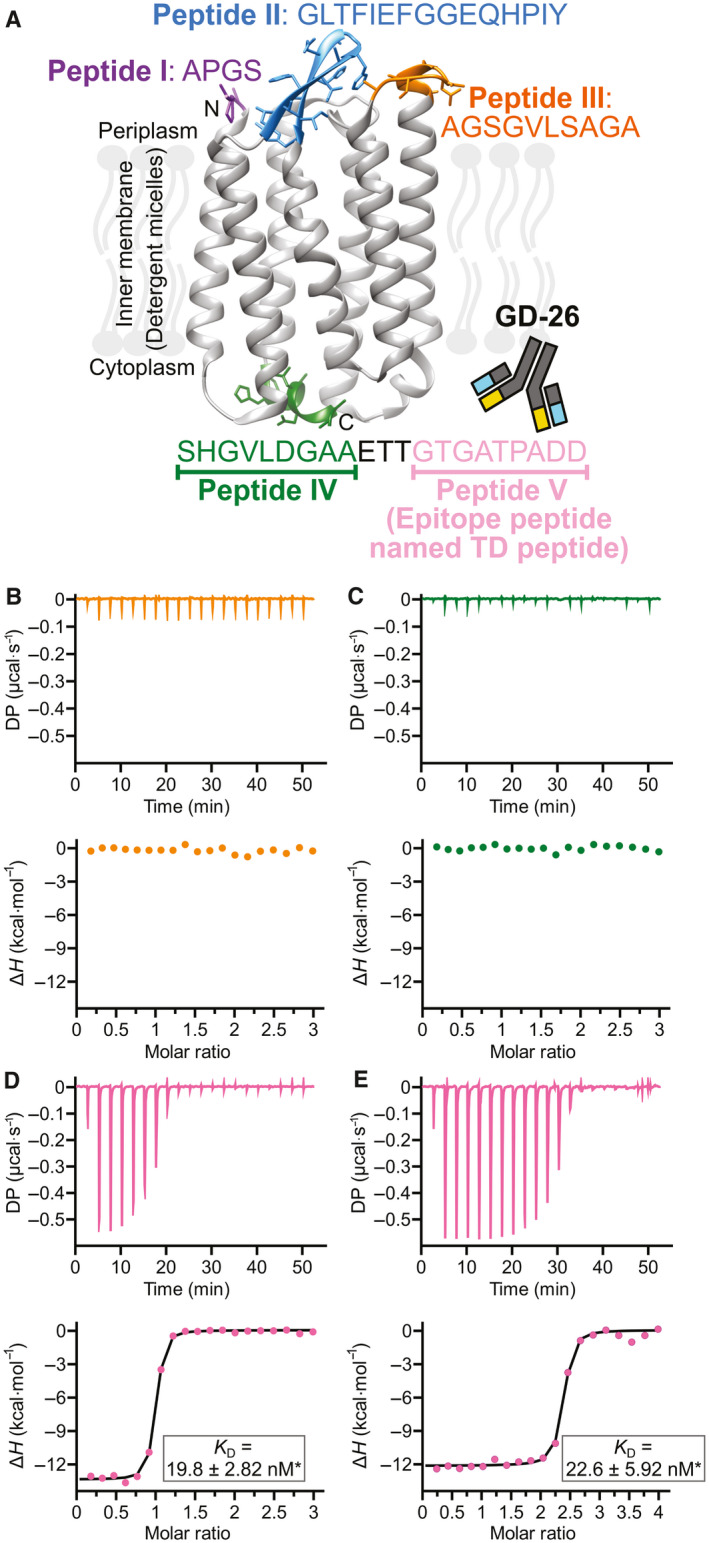
Recognition of *Hm*BRI D94N by GD‐26 Fab. (A) Illustration of the potential (peptides I–V) and the identified (pink, peptide V) epitope sites on *Hm*BRI D94N (PDB: 4PXK [23]) recognised by GD‐26 IgG. The structure of *Hm*BRI D94N (PDB: 4PXK [23]) shown here was generated by using the ucsf chimera software (University of California, San Francisco, CA, USA) [57]. The ITC binding profiles of (B) 150 μm peptide III and (C) 150 μm peptide IV with 10 μm GD‐26 Fab at 25 °C. The ITC binding profiles of peptide V (TD peptide) with (D) GD‐26 Fab and (E) GD‐26 IgG. The upper panels show the raw data of 150 μm synthetic TD peptide titrated into 10 μm GD‐26 Fab and 200 μm synthetic TD peptide titrated into 10 μm GD‐26 IgG at 25 °C. The lower panels show the binding isotherms plotted by integration of heat peaks against the molar ratio of TD peptide:GD‐26, displaying the experimental data points (●) and the best fit to the data (−). The best fit to the data of the TD peptide with GD‐26 Fab suggests *N* = 0.93 sites, *K*_D_ = 19.8 ± 2.82 nm*, ∆*H* = −13.4 kcal·mol^−1^, ∆*G* = −10.5 kcal·mol^−1^ and −*T*∆*S* = 2.90 kcal·mol^−1^. For the TD peptide with GD‐26 IgG, the best fit to the data suggests *N* = 2.28 sites, *K*_D_ = 22.6 ± 5.92 nm*, ∆*H* = −12.2 kcal·mol^−1^, ∆*G* = −10.4 kcal·mol^−1^ and −*T*∆S* *= 1.76 kcal·mol^−1^. *Errors shown here are the fitting errors of the linear fit to observed *K*_D_ from a single experiment (*n* = 1).

In the present study, we produced a recombinant monoclonal antibody named GD‐26 in mammalian cells as GD‐26 IgG was previously raised by immunising mice with *Haloarcula marismortui* bacteriorhodopsin I (*Hm*BRI) D94N mutant expressed in *Escherichia coli* (*E. coli*) and purified with detergents [21, 22]. Based on the published structure of *Hm*BRI D94N (PDB: 4PXK) [23], the potential epitope sites were predicted to be located at the loop regions or the C terminus (Fig. 1A). The synthetic peptides of these predicted epitopes (Fig. 1A, peptides III‐V) were studied by isothermal calorimetry (ITC) to determine whether there was a binding event taking place in the presence of the antigen‐binding fragment (Fab) of GD‐26. A nine amino acid peptide (GTGATPADD, named the TD peptide) located at the C terminus of *Hm*BRI D94N was determined to be the epitope for GD‐26 Fab, exhibiting a high affinity with a nanomolar (nm) dissociation constant *K*_D_. The crystal of GD‐26 Fab complexed with the TD peptide delivered a high‐quality structure at a resolution of 1.45 Å. Further epitope engineering was conducted based on the structure obtained for the GD‐26 Fab/TD peptide complex. By fusing the TD peptide to the C terminus of enhanced GFP (eGFP) as a model, the applications of the TD epitope tag were demonstrated by western blotting, ELISA and immunofluorescence as GD‐26 IgG successfully detected TD‐tagged eGFP. In summary, the GD‐26/TD peptide tagging system offers another tagging option for researchers as the TD tag adopts a unique 3_10_ helix structure and the protocol for the production of recombinant GD‐26 IgG and Fab is provided in this report.

## Results

### Identification of the epitope peptide for GD‐26 recognition

The novel monoclonal antibody GD‐26, identified as the mouse IgG2a isotype, was generated by immunising mice with *Hm*BRI D94N expressed in *E. coli*. In this study, GD‐26 Fab was expressed in a suspension‐adapted HEK293 cell line known as Expi293F. Based on the previously reported structure of *Hm*BRI D94N (PDB: 4PXK) [23] and other microbial bacteriorhodopsins [24, 25], it is known that *Hm*BRI D94N is a seven‐transmembrane protein whose transmembrane domains are linked by loops that are exposed at the surface. Being exposed to the solvent, the N terminus (Fig. 1A, peptide I), the loop regions (Fig. 1A, peptide II and peptide III) and the C terminus (Fig. 1A, peptide IV & peptide V) are hot spots for interactions with GD‐26 IgG. Although peptide V is not resolved in the existing *Hm*BRI D94N crystal structure (PDB: 4PXK) [23], it is an extension to peptide IV, which points outwards away from the transmembrane core. Five potential epitope sites were thus highlighted in Fig. 1A because the crystal structure of *Hm*BRI D94N (PDB: 4PXK) [23] suggests that these regions not only extend outwards but also own sufficient space for antibody binding without steric collisions. Peptide I at the N terminus (Fig. 1A) is so short that it is not very likely to be an epitope for antibody recognition [26]. Peptide II (Fig. 1A) exhibits a hairpin motif. The hairpin turn region, which is the most outward‐extending part of peptide II, consists of two glycine residues lacking side chains. Therefore, the likelihood of peptide II being a binding site for an antibody is low. Compared with peptides I and II, peptide III (Fig. 1A) is much more likely to be an antibody‐binding site due to its length, outward‐extending conformation and the presence of polar and hydrophobic residues. The C terminus was split into peptide IV and peptide V (Fig. 1A) because the most commonly found epitope length is around 10 amino acids [26]. Peptide IV exhibits an extended helix conformation consisting of polar and hydrophobic residues, while peptide V contains charged residues. Amino acid residues containing charged and/or hydrophobic side chains are more likely to interact with an antibody. Due to the secondary structure of peptide IV, the three amino acid residues (Glu, Thr and Thr) between peptides IV and V might serve as a linker in order to create space for peptide V for antibody binding. Alongside peptide III, peptide IV and peptide V were initially targeted as they showed a better chance of binding.

The three potential GD‐26‐binding sites on *Hm*BRI D94N (Fig. 1A, peptides III–V) were synthesised and investigated by studying their interactions with GD‐26 Fab using ITC (Fig. 1B–D). ITC measures the heat changes upon the binding of two molecules by titrating one interaction partner to the other until saturation (Fig. 1B–E: upper panels). These raw data are converted to a binding isotherm (Fig. 1B–E: lower panels) to be fitted to an appropriate binding model and to deliver comprehensive thermodynamic information of the studied interaction. The stoichiometry (*N*‐value) is determined as the x‐axis at the mid‐point (inflection point) of the sigmoidal fitting curve. The association constant *K*_A_ is derived from the slope at the inflection point, and the dissociation constant *K*_D_ is the reciprocal of *K*_A_. While peptide III and peptide IV showed no interaction with GD‐26 Fab (Fig. 1B,C), a considerable exothermic reaction (change in enthalpy Δ*H* = −13.4 kcal·mol^−1^) was observed upon injecting peptide V into GD‐26 Fab (Fig. 1D). It demonstrates that peptide V binds to GD‐26 Fab with a strong affinity as a dissociation constant *K*_D_ of 19.4 nm was measured with a 1 : 1 stoichiometry (*N* sites = 0.926). Peptide V (GTGATPADD, named TD peptide) is identified as the epitope peptide on *Hm*BRI D94N for the GD‐26 recognition. The TD peptide was recognised by GD‐26 IgG (Fig. 1E) with a similar affinity (*K*_D_ = 18.8 nm) but showing a 2 : 1 stoichiometry (*N* sites = 2.27), which indicates the divalency of GD‐26 IgG, in contrast to the 1 : 1 stoichiometry generated by monovalent GD‐26 Fab.

### Overall structure of GD‐26 Fab complexed with the TD peptide

**Fig. 2:**
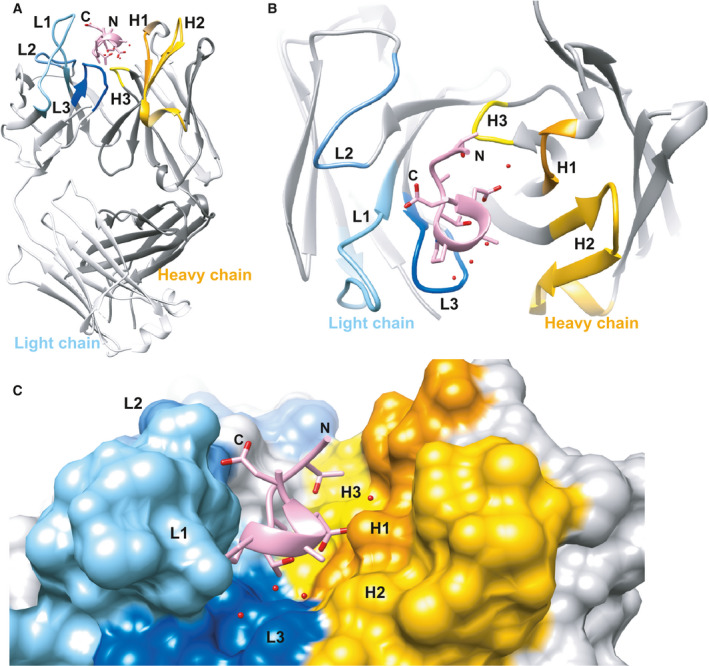
Overview of the structure of GD‐26 Fab complexed with TD peptide. (A) Side view of the complex structure illustrated as ribbon representation. The TD peptide is shown in pink, while the CDR regions of the heavy chain (H1–H3) and the light chain (L1–L3) of GD‐26 Fab are coloured in yellow and blue, respectively. (B) View from above the structure. (C) The surface representation of (A) with a zoomed‐in view of the antigen‐binding pocket. Water molecules are represented by red spheres. The cocrystal structure of GD‐26 Fab in complex with the TD peptide was generated by using the ucsf chimera software [57].

To understand the structural basis for the recognition of the TD peptide by GD‐26 as detected by ITC, GD‐26 Fab was cocrystallised with the TD peptide. The crystal structure of the GD‐26 Fab/TD peptide complex was determined by X‐ray crystallography, solved by the molecular replacement method, and eventually refined to a resolution of 1.45 Å (Table 1). GD‐26 Fab displays a canonical immunoglobulin fold consisting of antiparallel β‐sheets, which forms the constant and variable domains of the heavy and light chains (V_H_ and V_L_; Fig. 2A). All the complementarity‐determining region (CDR) loops contribute to the formation of the antigen‐binding pocket that holds the TD peptide in place (Fig. 2B,C).

**Table 1:** Data collection and refinement statistics.^a^

	GD‐26 Fab/TD peptide complex
Data collection	
Wavelength (Å)	1.0
Space group	*P*2_1_2_1_2_1_
Cell dimensions	
(Å)	*a* = 51.43, *b* = 74.34, *c* = 108.10
(°)	α = β = γ = 90.0
Resolution (Å)	25.0–1.45 (1.5–1.45)
Unique reflections	74 312
*R*_meas_ (%)	5.3 (55.7)
*I*/σ(*I*)	29.0 (3.0)
Completeness	99.9 (100.0)
Redundancy	5.6 (5.8)
CC1/2	0.97 (0.88)
CC*	0.99 (0.96)
Refinement	
Resolution (Å)	25.0–1.45
No. of reflections *R*_work_/*R*_free_	65 959/3767
*R*_work_/*R*_free_	13.0/17.8
No. of atoms/Avg *B* factor (Å^2^)	
Protein	3388/16.8
Water	755/34.7
RMSD	
Bond lengths (Å)	0.005
Bond angles (°)	0.83
Ramachandran statistics (%)^b^	
Favoured	98.60
Allowed	1.40
Outliers	0.00
Clash score	2.68
MolProbity score	1.13

CC* is a specific term used in X‐ray crystallography. It is a data quality indicator for crystallography.

^a^ Values corresponding to the highest resolution shell are shown in parentheses

^b^ The stereochemistry of the model was validated with MolProbity.

### Conformation of the TD peptide

**Fig. 3:**
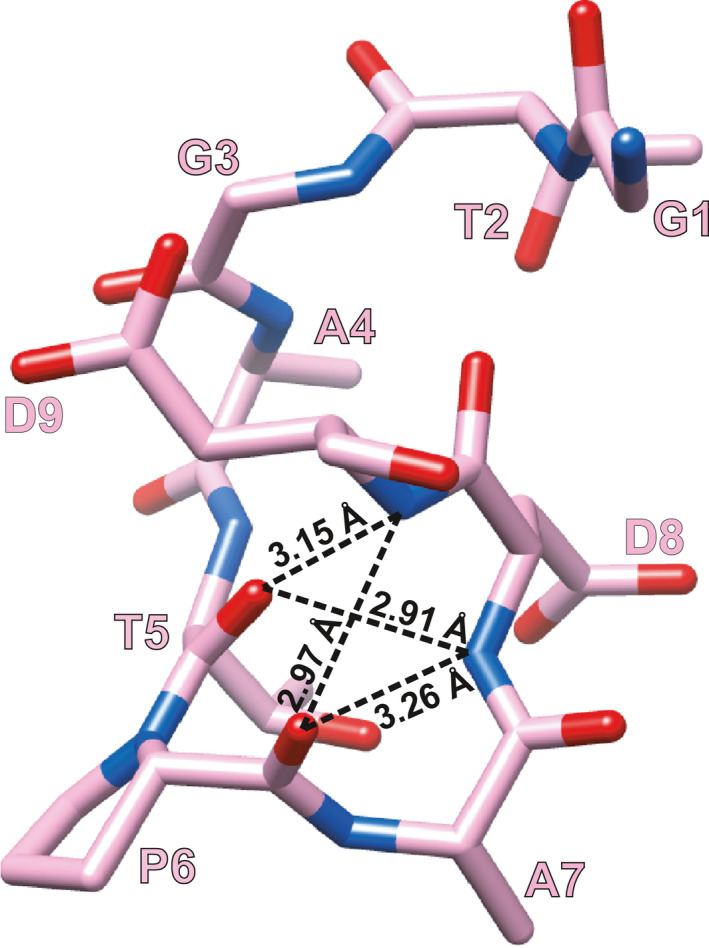
Structure of TD peptide. The TD peptide (GTGATPADD) is shown in stick representation with carbons coloured in pink, oxygens in red and nitrogens in blue. Intrapeptide hydrogen bonds (distance less than 3.5 Å) are indicated by the dashed lines with distances measured. The TD peptide structure was generated by using the ucsf chimera software [57].

The TD peptide found in the GD‐26 Fab/TD peptide complex adopts a compact 3_10_ helix conformation mainly comprised of residues Pro6, Ala7 and Asp8 (Fig. 3). This short 3_10_ helix is formed and stabilised by hydrogen bonds attributed to the carbonyl and amino groups of the backbone. The 3_10_ helix is buried deepest in the antigen‐binding pocket between the V_H_ and V_L_ interface, while the N terminus and the C terminus of the TD peptide both exhibit an extended structure towards the surface of GD‐26 Fab (Fig. 2A,C).

### Interactions between GD‐26 Fab and the TD peptide

**Fig. 4:**
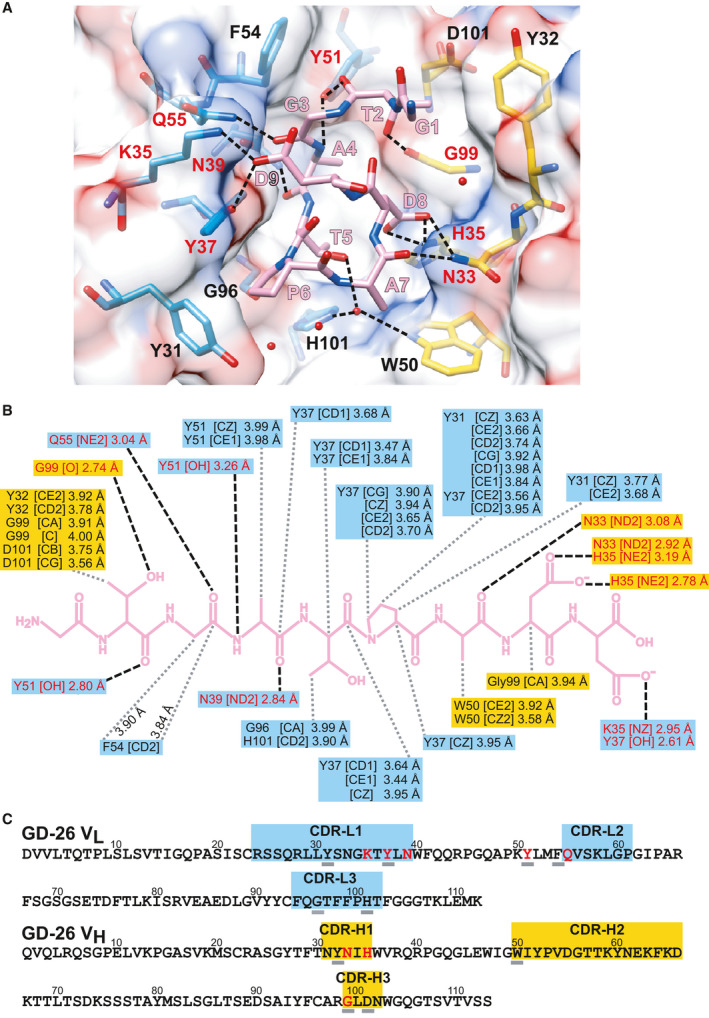
Interactions between GD‐26 Fab and TD peptide. The polar (black dashed lines) and nonpolar (grey dotted lines) interactions between GD‐26 Fab (V_L_ residues in blue and V_H_ in yellow) and the TD peptide (pink) are indicated. The amino acid residues of GD‐26 Fab highlighted in red are involved in polar interactions. (A) GD‐26 Fab and the TD peptide are shown in stick representation with oxygens coloured in red and nitrogens in blue. Surface representation shows the electrostatic surface potential of GD‐26 Fab with negative charges in red and positive charges in blue. Water molecules are represented by red spheres. The structure of the GD‐26 Fab/TD peptide complex was generated by using the ucsf chimera software [57]. (B) Summary of the polar and nonpolar interactions in the GD‐26 Fab/TD peptide complex (distances in Å). (C) The variable domain amino acid sequences of GD‐26. The coloured boxes indicate CDRs 1–3 (IMGT definition [66]). Amino acids that are underlined are involved in nonpolar interactions as indicated by the grey dotted lines in (B).

In addition to the hydrogen bonds formed by the TD peptide backbone as mentioned above, there is a network of hydrogen bonds contributed by the side chains of the TD peptide and the residues of the surrounding CDR regions (Fig. 4). The side chains of Asp8 and Asp9 of the TD peptide form charge‐assisted hydrogen bonds with the side chains of His35 in CDR‐H1 and Lys35 in CDR‐L1, respectively (Fig. 4A,C). Moreover, the side chain of Thr5 of the TD peptide forms water‐mediated hydrogen bonds with the side chains of Typ50 in CDR‐H2 and His101 in CDR‐L3. The backbone carbonyl groups of Thr2, Gly3, Ala4 and Ala7 of the TD peptide make hydrogen bonds with the side chains of Tyr51, Gln55 and Asn39 in V_L_ and Asn33 in V_H_. The side chain of Tyr51 in V_L_ is also hydrogen‐bonded to the backbone amino group of Ala4 of the TD peptide.

Extensive nonpolar interactions (Fig. 4B) also contribute to the high‐affinity binding interaction between GD‐26 Fab and the TD peptide, along with the hydrogen bond contacts. The position of Pro6 in the TD peptide, the start of the 3_10_ helix, shows that Pro6 is involved in face‐to‐face π stacking with Tyr37 and edge‐to‐face π stacking with Tyr31 in CDR‐L1 (Fig. 4A). The CH/π interactions are also present between the backbone of the TD peptide and Tyr31, Tyr37, Tyr51 and Phe54 in V_L_ and Trp50 in V_H_.

### Engineering of the TD peptide

Despite all the thermodynamic parameters indicated in the preliminary result of the TD peptide binding to GD‐26 Fab (Fig. 1D), the *K*_D_ for the TD peptide with GD‐26 Fab was determined as 12 ± 2.8 nm (mean ± SEM) from four independent experiments (*n* = 4; Table 2). Located at the end of the TD peptide, Asp9 is involved in the binding to V_L_ of GD‐26 Fab (Fig. 4). Substituting Asp9 with the uncharged isosteric residue Asn resulted in a reduced affinity due to the loss of charge‐assisted hydrogen bonds (Table 2, peptide D9N). Removing Asp9 (Table 2, peptide C1) from the TD peptide caused the further loss of interactions with GD‐26 Fab, while depleting both Asp9 and Asp8 (Table 2, peptide C2) led to a complete loss of binding to GD‐26 Fab. Truncation of the C terminus of the TD peptide benefited neither specificity nor enhanced binding as there was a substantial decrease in the *K*_D_ from 12 to 548 nm that resulted from the deletion of both Gly1 and Thr2 (Table 2, peptide N2). Further deletion of Gly3 and Ala4 completely diminished its capability to bind to GD‐26 Fab (Table 2, peptide N4) even though the N terminus remained intact. The results suggest that the ideal length of the antigen peptide for the recognition by GD‐26 Fab remains as nine amino acids (Table 2, blue zone), ensuring enough effective contacts within the antigen‐binding pocket. The residues at the N terminus and the C terminus of the TD peptide show the importance of collective hydrogen bonds and ionic interactions in the binding to GD‐26 Fab.

**Table 2:** Changes in the binding to GD‐26 Fab due to engineering of TD peptide. Amino acid replacement based on the wild‐type TD peptide is indicated in red. The strength of interactions between GD‐26 Fab and the engineered peptides is described as *K*_D_. The peptides coloured in blue study the minimal and ideal length of an antigen peptide for GD‐26 Fab. The peptides coloured in yellow investigate whether minimising the unoccupied space around Ala7 can enhance the binding to GD‐26 Fab. The peptides coloured in pink study the importance of π–π interactions at peptide position 6. The peptides coloured in grey test whether the TD peptide can act as an N‐terminal tag.

Peptide	Sequence	*K*_D_ (nm)
TD	**G**TGAT**PA**DD	12 ± 2.8^a^
D9N	GTGATPAD **N**	132
C1	GTGATPAD	273
C2	GTGATPA	No binding
N2	GATPADD	548
N4	TPADD	No binding
A7W	GTGATP **W** DD	34.4
A7E	GTGATP **E** DD	46.4
A7Q	GTGATP **Q** DD	59.3
A7S	GTGATP **S** DD	72.7
A7Y	GTGATP **Y** DD	73.4
A7H	GTGATP **H** DD	94
A7R	GTGATP **R** DD	171
A7T	GTGATP **T** DD	449
A7P	GTGATP **P** DD	1040
P6F	GTGAT **F** ADD	78.2
P6A	GTGAT **A** ADD	140
P6G	GTGAT **G** ADD	892
G1M	**M** TGATPADD	46.5
M1	**M** GTGATPADD	25.1

^a^ The *K*_D_ for the wild‐type TD peptide interacting with GD‐26 Fab is mean ± SEM determined (*n* = 4), while the rest of the *K*_D_ values in this table is from a single experiment (*n* = 1).

Based on the structural information on GD‐26 Fab complexed with the TD peptide (Fig. 4), we noticed that there was unoccupied space around Ala7. By modelling, amino acids that could potentially replace Ala7 and still fit into the antigen‐binding pocket were predicted. Synthetic peptides with predicted substitutions were mixed with GD‐26 Fab on ITC to monitor changes in *K*_D_ (Table 2, yellow zone). The resulting *K*_D_ values showed that the substitutions of Ala7 on the TD peptide failed to improve the affinity for GD‐26 Fab.

The special position of Pro6 in the centre of the TD peptide was investigated as well. Substitution of Pro6 to Ala and Gly both resulted in a weaker interaction (Table 2, pink zone). Interestingly, when Pro6 was substituted by Phe, which contains a benzene ring, the affinity was partially recovered. This is in agreement with the π‐π stacking interactions observed within the antigen‐binding pocket where Pro6 on the TD peptide interacts with Tyr31 and Tyr37 in CDR‐L1 through π stacking (Fig. 4A).

A residue of Met was attached to the N terminus of the TD peptide to test the applicability of the TD peptide as an N‐terminal epitope tag for protein expression (Table 2, grey zone). The peptide G1M and peptide M1 both showed a strong interaction with GD‐26 Fab as indicated by the *K*_D_ values (46.5 nm and 25.1 nm, respectively), which are comparable with the *K*_D_ of 12 nm obtained from the TD peptide without the added Met residue, indicating the application of the TD peptide as an N‐terminal tag in addition to the C‐terminal tag originated from *Hm*BRI D94N. Since the first trials of amino acid replacement stated above did not achieve a greater affinity than the wild‐type TD peptide, it was decided not to repeat these trials due to time and cost constraints. Thus, the *K*_D_ values in Table 2 are from a single experiment (*n* = 1) without SEM apart from the *K*_D_ for the wild‐type TD peptide.

### Applications of the GD‐26/TD tagging system

**Fig. 5:**
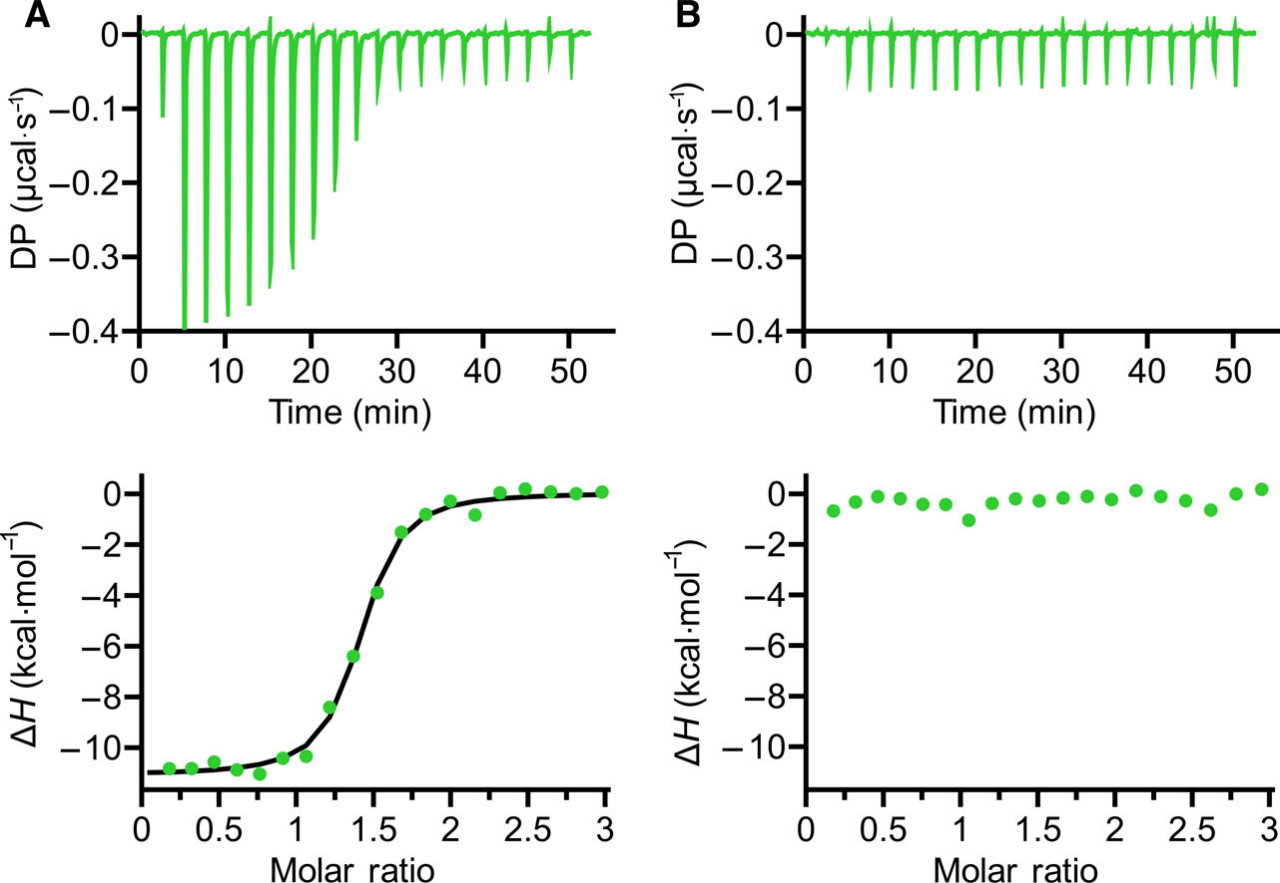
Interaction of GD‐26 Fab with TD‐tagged eGFP and untagged eGFP measured by ITC at 25 °C. (A) The ITC binding profiles of 155 μm TD‐tagged eGFP titrated into 10 μm GD‐26 Fab. The best fit to the data suggests *N* = 1.35 sites, *K*_D_ = 129 ± 19.2 nm*, ∆*H* = −11.1 kcal·mol^−1^, ∆*G* = −9.4 kcal·mol^−1^ and −*T*∆*S* = 1.73 kcal·mol^−1^. (B) The ITC binding profile of 9.57 μm GD‐26 Fab with 142 μm untagged eGFP. *Errors shown here are the fitting errors of the linear fit to observed *K*_D_ from a single experiment (*n* = 1).

**Fig. 6:**
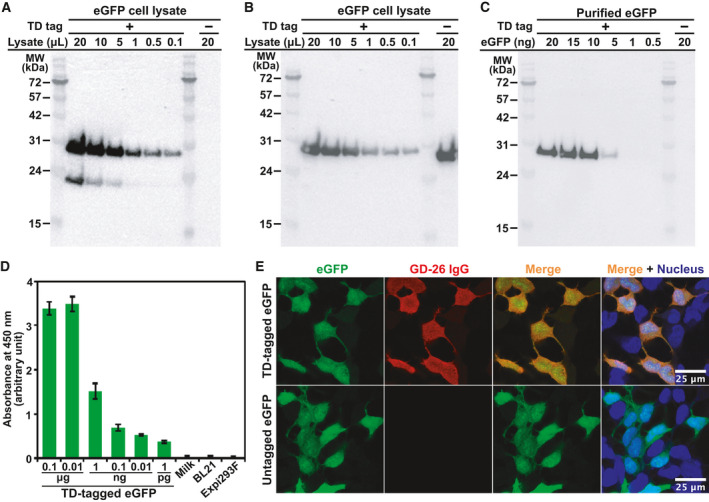
Applicability of the GD‐26/TD peptide tagging system. Application of the TD peptide tag onto the C terminus of eGFP (˜ 28 kDa) as a demonstration model (a polyhistidine tag is present at the N terminus). Western blot analysis of *Escherichia* *coli* cell lysates expressing eGFP tagged with and without the TD tag detected by (A) GD‐26 IgG and (B) anti‐histidine tag antibody applied as the primary antibody. (C) Western blot analysis of purified TD‐tagged eGFP (0.5–20 ng) detected by GD‐26 IgG. (D) The sensitivity of GD‐26 IgG (1 : 10 000) was tested against purified TD‐tagged eGFP (0.1 µg–1 pg) in ELISAs. The binding results were displayed as bar charts of the mean absorbance values at 450 nm of triplicate determinations with the standard deviation errors (*n* = 3) shown. Controls consisting of the uncoated wells (milk) and wells coated with cell lysates of BL21(DE3) and mammalian cell Expi293F are included. (E) Detection of TD‐tagged eGFP by immunofluorescence. The upper panels show HEK293 cells transfected with TD‐tagged eGFP, and the lower panels show HEK293 cells transfected with eGFP without the TD tag. Both were fixed, incubated with GD‐26 IgG and stained by Alexa Fluor 568 goat anti‐mouse IgG (H + L) secondary antibody (Thermo Fisher Scientific). Left to right: intrinsic eGFP signal; Alexa Fluor 568‐bound GD‐26 IgG; overlay; overlay including DAPI‐stained nuclei. Scale bar = 25 µm.

Short epitope peptides and paired monoclonal antibodies have the potential to be developed into a powerful tagging system for protein purification and detection. We tested the possibility and feasibility of the TD peptide and GD‐26 IgG to be a tagging system. First, a short linker consisting of two glycine residues and the TD peptide were fused to the reporter protein eGFP at the C terminus, while a polyhistidine tag was placed at the N terminus of eGFP. GD‐26 IgG was able to recognise not only the synthetic TD peptide (Fig. 1E) but also TD‐tagged eGFP expressed in *E. coli* with a *K*_D_ value of 129 nm derived from ITC (Fig. 5). Downstream applications of the GD‐26/TD tagging system were studied. Western blotting showed specific and reliable detection of GD‐26 IgG to TD‐tagged eGFP (∼ 28 kDa) in crude *E. coli* cell lysates (Fig. 6A). An additional low molecular weight band below TD‐tagged eGFP was also detected by GD‐26 IgG in Fig. 6A. As mentioned above, a polyhistidine tag was fused to the N terminus of TD‐tagged eGFP and this lower band was no longer observed in western blotting using the anti‐polyhistidine antibody for detection (Fig. 6B). Therefore, the additional band below TD‐tagged eGFP in Fig. 6A represents the N‐terminal degradation product of TD‐tagged eGFP whose TD peptide at the C terminus remains intact for detection by GD‐26 IgG. Besides cell lysates, as little as 5 ng of purified TD‐tagged eGFP was detected by GD‐26 IgG using western blotting (Fig. 6C), while eGFP without the TD peptide showed no detection signals as expected (Fig. 6A,C). In addition to western blotting, ELISA revealed that GD‐26 IgG was able to detect purified TD‐tagged eGFP at picogram level (Fig. 6D). Instead of coating with TD‐tagged eGFP, the uncoated wells and the wells coated with cell lysates of *E. coli* BL21(DE3) and mammalian cell Expi293F showed negligible absorbance at 450 nm, indicating that GD‐26 IgG detection to TD‐tagged eGFP is sensitive and specific.

We also generated a human construct in which the TD peptide was fused to the C terminus of eGFP to be expressed in HEK293 cells. This is to validate whether the GD‐26/TD peptide tagging system can be utilised in cell lines that are capable of post‐translational modifications. In immunofluorescence applications on transfected and then PFA‐fixed HEK293 cells (Fig. 6E), GD‐26 IgG specifically recognised TD‐tagged eGFP (Fig. 6E, red) and the signal colocalised with the intrinsic eGFP signal (Fig. 6E, green). eGFP without the TD peptide remained its intrinsic signal in HEK293 cells, but it showed no GD‐26 IgG signal as untagged eGFP did not contain the TD peptide tag for GD‐26 IgG to bind to. Characterising the interactions between the monoclonal antibody GD‐26 and the TD peptide contributes to employment of this novel peptide tag in scientific research.

## Discussion

**Fig. 7:**
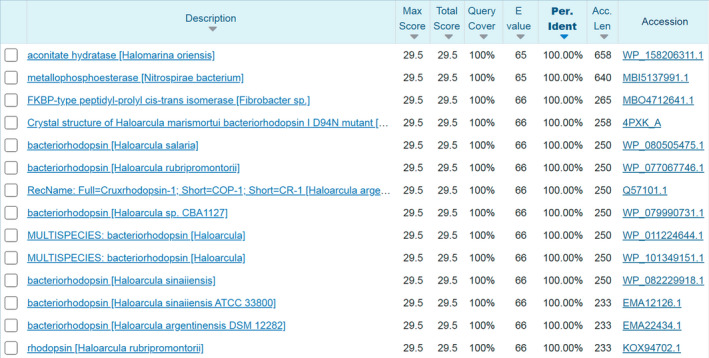
Protein BLAST compares the TD peptide sequence (GTGATPADD) with the database of protein sequences. The BLAST results for the TD peptide at 100% coverage and 100% similarity. (https://blast.ncbi.nlm.nih.gov/Blast.cgi?PAGE=Proteins).

As a by‐product while developing antibodies against the membrane protein *Hm*BRI D94N, a novel epitope tagging system comprising the TD peptide (GTGATPADD) and GD‐26 monoclonal antibody was established. Taking the archaeal origin of the TD peptide into account, the protein BLAST analysis (Fig. 7) shows lack of identical sequences within proteins of *E. coli* and eukaryotic cells, which are common recombinant protein expression systems. The uniqueness makes the TD peptide a potentially advantageous epitope tag. The affinity, specificity and structural basis for the interactions between the TD peptide and GD‐26 Fab, as well as the applications of GD‐26 IgG and the TD tag, are characterised in the present study.

The crystal structure of GD‐26 Fab complexed with the TD peptide revealed that the TD peptide adopted a 3_10_ helix‐like structure (Figs 2 and 3) embedded in a pocket lined by aromatic and side‐chain amide residues in the interface between the heavy and light chains on the surface of GD‐26 Fab (Fig. 4A). A proline residue is frequently found in the formation of a 3_10_ helix in a short peptide [27, 28, 29]. The unique cyclic conformation of the TD peptide has a propensity to form before encountering GD‐26 Fab, attributed to the intrapeptide hydrogen bonds and the presence of proline located at position 6 which is the start of the 3_10_ helix. Similar to the structure reported in this study, recognition of the CD20 epitope by rituximab [27] shows that CD20 forms a 3_10_ helix whose proline residue is also present at the turn of the helix and located at the bottom of the binding cleft. Our Fab–peptide complex structure has also complemented an earlier publication, which reports a crystal structure of *Hm*BRI D94N (PDB: 4PXK) with an undetermined C‐terminal region due to disorder [23].

The presence of the 3_10_ helical conformation is critical for the recognition of the TD peptide by GD‐26 Fab as the extended‐chain conformation is unlikely to fit into the same binding cleft generated by GD‐26 Fab due to shape complementarity and spatial restrictions [30]. The high affinity of this interaction described by a *K*_D_ of 12 nm is explained by the structural basis exhibiting hydrogen bonds and π‐stacking (Fig. 4) to anchor the bound peptide ligand. First, we found multiple aromatic residues in the binding cleft, especially Tyr31 and Tyr37 in CDR‐L1, which adopt edge‐to‐face and face‐to‐face π stacking with the side chain of Pro6 in the TD peptide to stabilise the binding. Prolines are known to play a role in protein–protein and protein–peptide interactions with aromatic residues from the other molecule via favourable hydrophobic effects, C–H∙∙∙π and C–H∙∙∙O interactions [31, 32, 33, 34]. Secondly, both the N terminus and C terminus of the TD peptide greatly contribute to the specificity and high affinity for the recognition by GD‐26 Fab (Table 2). Aspartic acids are among the most common hydrogen acceptors in charged hydrogen bonds [35, 36]. Asp8 and Asp9 in the TD peptide interact with CDR‐H1 and CDR‐L1, respectively, via charge‐assisted hydrogen bonds to dominate the binding. In addition to threonine and aspartic acid residues, the TD peptide also contains two glycine residues at positions 1 and 3. Glycine residues may have an advantage of providing backbone flexibility to the peptide antigen to better satisfy spatial constraints within the binding cleft due to lack of side chains [35]. As a result, a network of polar and nonpolar interactions gives rise to the compact folding of the TD peptide, the high affinity and the specificity between GD‐26 Fab and the TD peptide.

In terms of specificity, various amino acid substitutions in the TD peptide retain the ability to interact with GD‐26 Fab despite the reduced affinities (Table 2). Substitution of Pro6 to Phe instead of Gly and Ala shows a recovered affinity. This indicates the importance of the π‐stacking interactions at the position 6 of the TD peptide, while Pro remains as the ideal residue at this position in terms of the residue size and side‐chain orientation for both edge‐to‐edge and edge‐to‐surface π interactions. We have characterised the interaction between the TD peptide and GD‐26 Fab both structurally and compositionally.

Compared with the synthetic TD peptide that is freely accessible in solution and binds to GD‐26 Fab with a *K*_D_ of 12 nm (Table 2), TD‐tagged eGFP expressed in *E. coli* binds to GD‐26 Fab with a *K*_D_ of 129 nm (Fig. 5). The decrease in the affinity observed may be caused by the linker between eGFP and the TD peptide. The TD peptide was fused to the C terminus of eGFP with a short linker composed of two glycine residues, which are flexible amino acids due to lack of side chains. This short linker is aimed at providing minimal space for the TD peptide to avoid collision with the linked proteins. The accessibility of the TD peptide tag may be hindered by the adjacent amino acids and the protein structure it is inserted into, leading to a reduced affinity. Despite the low affinity for TD‐tagged eGFP and GD‐26 Fab, the ELISA results (Fig. 6D) showed that GD‐26 IgG was able to detect the coated antigen as low as 1 pg in concentration. This strong sensitivity is most likely attributed to the signal amplification from the HRP‐conjugated secondary antibody, as well as the avidity arising from the bivalency of GD‐26 IgG. When antigens (eGFP) are tethered to a surface in sufficiently close proximity, the two Fab binding sites of GD‐26 IgG can be simultaneously occupied leading to an increase in total binding strength, hence enhanced affinity and stability [37, 38] as compared to the monovalent affinity given by GD‐26 Fab. The interactions between TD‐tagged eGFP and GD‐26 IgG are stable enough to withstand extensive washing steps and to be detected in western blotting, ELISA and immunofluorescence (Fig. 6) without cross‐reactivity, suggesting the effectiveness of the GD‐26/TD peptide tagging system. Importantly, the TD peptide seems compatible with the folding of the tagged proteins as eGFP remains its intrinsic fluorescent property of the chromophore contained within the β‐barrel fold (Fig. 6E). The 3_10_ helical structure of the TD peptide is involved in the interactions with GD‐26 Fab (Fig. 2). Western blotting results on TD‐tagged eGFP (Fig. 6A,C) in the presence of GD‐26 IgG suggest that after SDS/PAGE, the TD peptide retains its 3_10_ helical structure for detection by GD‐26 IgG. SDS is an anionic detergent that binds to proteins primarily through hydrophobic interactions to denature and make proteins negatively charged for electrophoresis. (For our sample preparation, we used lithium dodecyl sulfate, which is identical to SDS apart from the salt.) Proteins treated by SDS adopt an extended form. Instead of unfolding proteins completely, SDS tends to aggregate at hydrophobic sites generating an extended polypeptide chain containing SDS‐coated helical regions connected by uncoated segments [39, 40]. Considering that SDS molecules do not bind to nor linearise all regions of a protein uniformly, the region of TD peptide is likely to keep its 3_10_ helical structure in SDS/PAGE since it seems more difficult for SDS molecules to interact with the TD peptide due to the hydrophilic property and net negative charge of the TD peptide. Besides, the proline residue within the TD peptide may also contribute to the conformational rigidity preserving the 3_10_ helix in the presence of SDS [41].

General requirements for epitope tags are small size, being monomeric, good solubility and stability and, importantly, little structural and functional effects on the fused proteins. Being isolated from the membrane protein *Hm*BRI, the TD peptide has a natural amino acid sequence that is not found in any endogenous proteins of *E. coli* nor mammalian cells (Fig. 7). It is not surprising that the TD peptide has good water solubility since it is originated from the cytoplasmic tail of *Hm*BRI and has the net negative charge at pH 7.0. The TD peptide also shows good cellular protease resistance and proteolytic stability. Without using protease inhibitors, the TD peptide remains attached and detectable after protein expression and purification from bacterial and mammalian cell systems (Fig. 6). Another advantage of using the TD peptide tag is that GD‐26 IgG is specific without cross‐reactivity in both bacterial and mammalian lysates (Fig. 6). Besides, GD‐26 IgG has a long‐term (over a year) stability stored at 4 °C in Tris buffer. The GD‐26/TD peptide tagging system possesses all the above‐mentioned features such as other already‐existing epitope tags such as the FLAG and c‐Myc tags do. The affinity of GD‐26/TD peptide at nanomolar level is also comparable with those commonly used epitope tags (Table 3). What makes the TD peptide stand out from other epitope tags is that it is derived from a membrane protein and its interactions with GD‐26 IgG are compatible with detergents, similar to the 1D4 tag [42]. The helical propensity of proline has been previously reported to be enhanced in the presence of detergent micelles [43], which may apply to the presence of proline within the TD peptide. Moreover, our TD peptide is one of those rare epitope tags that holds a specific structure in solution (i.e. the ALFA tag [2]) rather than an extended conformation found in most of the existing epitope tags including the FLAG, c‐Myc, HA and polyhistidine tags.

**Table 3:** Affinities and structures of common epitope tags.

Epitope tag	Sequence	Anti‐tag antibody	Affinity (*K*_D_)	Structure (PDB ID)
TD	GTGATPADD	GD‐26	12 ˜ 129 nm	7DOH
FLAG	DYKDDDDK	M2	3 ˜ 31 nm [46, 59, 60]	2G60 [61]
HA	YPYDVPDYA	4B2	1.6 nm [46]	No data available
c‐Myc	EQKLISEEDL	9E10	2.2 ˜ 560 nm [46, 62, 63]	2OR9 [64]
Polyhistidine	6–12 Histidines	3D5	No data available	1KTR [45]
ALFA	SRLEEELRRRLTE	Nanobody ALFA	26 pm [2]	6I2G [2]
PA	EGGVAMPGAEDDVV	NZ‐1	1.8 pm ˜ 0.4 nm [46]	4YO0 [47]
DLVPR	DLVPR	G196	1.25 nm [65]	4YO0 [47]

Not every epitope tagging system has the crystal structures solved. Crystal structures of the following epitope tags complexed with either Fab or scFV fragments are reported: the c‐Myc tag [44], the polyhistidine tag [45], the PA tag [5, 46, 47], the P20.1 system [48], the PA tag [5, 47], the SpyTag [49] and the ALFA tag [2] (Table 3). The published structures show that the majority of the epitope peptides adopt an extended conformation. Nevertheless, the PA tag adopts a U‐shaped conformation upon binding and this characteristic allows the PA tag to replace the loop region of the protein of interest so that anti‐PA Fab can act as a crystallisation chaperone [5, 47].

The ALFA tag forms a stable α‐helical structure itself, and ALFA‐tagged proteins are recognised by a specialised nanobody for downstream applications such as immunoprecipitations and super‐resolution microscopy [2]. One of the most important considerations in choosing epitope tags is the downstream applications of the tagged protein. Recently, grafting the key residues of a donor helix onto an exposed acceptor helix of the target protein has been demonstrated and validated as a powerful tool for X‐ray crystallography and electron microscopy in combination with the use of off‐the‐shelf anti‐helix (donor helix) antibodies [50]. This epitope grafting approach facilitates electron microscopy studies by increasing the size of a target protein over 50 kDa, commonly known as the detection limit, without the need to develop target‐specific antibodies. Since the 3_10_ helix exhibits a rigid conformation for the recognition by GD‐26 Fab, we anticipate that our GD‐26/TD peptide tagging system may be applied to X‐ray crystallography and electron microscopy as a crystallisation chaperone by inserting the TD peptide into the loop/turn region of a target protein. To our knowledge, our GD‐26/TD peptide tagging system utilises the intrinsic short 3_10_ helical structure, which is not observed in other tags to achieve a structural feature that remains structured in applications such as SDS/PAGE.

We offer a novel epitope tag characterised by a high‐affinity interaction between the 3_10_ helix‐forming TD peptide and GD‐26 IgG, adding to the current list of choices. As 3_10_ helices are commonly found in proteins including targets for anticancer [27], antiviral [28, 29] and Alzheimer's disease [30] studies, the structural basis for GD‐26 Fab complexed with the TD peptide followed by peptide engineering provides a better understanding of this high‐affinity interaction and may contribute to other structural studies involving antibodies and 3_10_ helices.

## Materials and methods

### Expression and purification of GD‐26 IgG and GD‐26 Fab

**Fig. 8:**
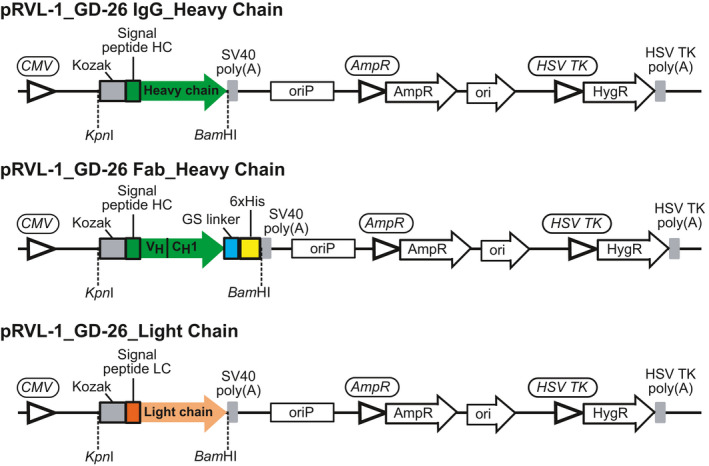
Design of expression constructs for GD‐26 IgG and Fab production. Illustration of the expression constructs designed to code for GD‐26 IgG (pRVL‐1_GD‐26 IgG) and GD‐26 Fab (pRVL‐1_GD‐26 Fab) in mammalian cells. The signal peptide HC (MDWTWRVFCLLAVAPGAHS) is derived from human immunoglobulin heavy variable 1–46 (IGHV1‐46). The signal peptide LC (MVLQTQVFISLLLWISGAYG) is derived from human immunoglobulin kappa variable 4‐1 (IGKV4‐1).

The monoclonal antibody GD‐26 was generated by immunising mice with *Hm*BRI D94N expressed in *E. coli* and then sequenced (GenScript, Piscataway, NJ, USA). The plasmid constructs of GD‐26 IgG and GD‐26 Fab (Fig. 8) were generated based on the previous publication [51] utilising transient expression in mammalian cells Expi293F (Thermo Fisher Scientific, Waltham, MA, USA). The genes encoding the heavy and light chains were synthesised (GenScript) and subcloned into the pRVL‐1 plasmid (Addgene, Watertown, MA, USA) [51] digested with *Kpn*I and *Bam*HI restriction enzymes (Fig. 8). All plasmids were amplified using *E. coli* DH5α strain and purified using EasyPrep EndoFree Maxi Plasmid Extraction Kit (BIOTOOLS, New Taipei City, Taiwan).

Expi293F cells were transfected following the manufacturer's instructions. To make GD‐26 IgG, cotransfection of two plasmids, pRVL1_GD‐26 IgG_Heavy Chain and pRVL1_GD‐26_Light Chain (Fig. 8), was made at a 1 : 2 ratio. Likewise, to make GD‐26 Fab, cotransfection of pRVL1_GD‐26 Fab_Heavy Chain and pRVL1_GD‐26_Light Chain (Fig. 8) was made at a 1 : 2 ratio. Six days after transfection, the medium was collected because the antibody and Fab were secreted. The medium containing GD‐26 IgG was diluted with the Tris buffer (50 mm Tris, 150 mm NaCl, pH 7.5) at a 1 : 1 ratio and applied onto a HiTrap Protein G HP column (GE Healthcare, Chicago, IL, USA). GD‐26 IgG was eluted with the glycine/HCl buffer (0.1 m, pH 2.5) and dialysed against the Tris buffer for further purification by gel filtration chromatography on a Superdex 200 Increase 10/300 GL column (GE Healthcare) to remove any aggregates. The medium containing GD‐26 Fab was diluted with the Tris buffer (50 mm Tris, 150 mm NaCl, pH 7.5) at a 1 : 1 ratio and applied onto a HisTrap Excel column (GE Healthcare). GD‐26 Fab was eluted with the Tris buffer supplemented with 500 mm imidazole and then further purified by gel filtration chromatography on a Superdex 200 Increase 10/300 GL column (GE Healthcare).

### Expression and purification of TD peptide‐tagged eGFP

The eGFP gene encoding residues 1–231 with eight histidines at the N terminus and the TD peptide with a linker consisting of two glycine residues at the C terminus was synthesised and subcloned into the pET11a plasmid digested with *Nde*I and *Bam*HI restriction enzymes (BIOTOOLS). eGFP was expressed in *E. coli* BL21(DE3) in Luria–Bertani medium containing 100 µg·mL^−1^ ampicillin. When reaching an OD600 value of 0.6, cells were induced with 1 mm IPTG and incubated overnight at 20 °C. Following expression, cell pellets were dissolved in the Tris buffer (50 mm Tris, 500 mm NaCl, pH 7.5) and disrupted by a NanoLyzer N2 homogeniser (Gogene Corporation, Xinpu, Hsinchu County, Taiwan). After applying the lysates to ultracentrifugation (Beckman Type 45 Ti rotor, Beckman Coulter, Brea, CA, USA) at 186 000 ***g*** for 1.5 h at 4 °C, the supernatant was applied onto a HisTrap Excel column (GE Healthcare). The column was washed by the Tris buffer supplemented with 20, 50 and 100 mm imidazole and then eluted by 500 mm imidazole. Eluted eGFP was further purified by gel filtration chromatography on a Superdex 200 Increase 10/300 GL column (GE Healthcare) with the use of the Tris buffer (50 mm Tris, 150 mm NaCl, pH 7.5) to remove aggregates and imidazole.

### Isothermal calorimetry PEAQ‐ITC

The titration experiments were performed with a MicroCal PEAQ‐ITC Automated (Malvern Panalytical, Malvern, UK) at 25 °C. The ITC sample cell contained 200 μL of GD‐26 IgG or GD‐26 Fab in the Tris buffer (50 mm Tris, 150 mm NaCl, pH 7.5). In the syringe, 40 μL of synthetic peptides (peptide III–V) or TD‐tagged eGFP in the same Tris buffer was injected into the sample cell using 20 injections (0.4 μL for the first injection). Prior to data analysis, heats of dilution generated by injecting the peptide solution into the buffer in the sample cell were subtracted from the experimental data. The experimental raw data were analysed using the MicroCal PEAQ‐ITC Analysis software package provided with the instrument. The first data point was removed for fitting the data to a one‐set‐of‐sites model. After entering sample concentrations, the inbuilt function for fitting is operated by clicking iteration button until a good fit to the experimental data points of the isotherms is achieved and Chi‐squared is no longer decreasing. The final fitting parameters including *K*_D_, changes in enthalpy (Δ*H*), entropy (Δ*S*) and Gibb's free energy (Δ*G*) and binding stoichiometry were generated by the same software.

### Crystallisation, data collection, structure determination and refinement

Purified GD‐26 Fab (20.8 mg·mL^−1^) was mixed with the synthetic TD peptide at a molar ratio of 1 : 6 for 1 h at room temperature. The GD‐26 Fab/TD peptide complex crystal was grown by mixing 0.3 µL of the mixture with 0.3 µL reservoir solution using the sitting drop vapour diffusion method at room temperature. The crystals were grown at 18 °C and obtained in 25% (w/v) PEG 3350, 0.2 m ammonium acetate, and 0.1 m Bis‐Tris, pH 5.5.

XRD experiments were carried out at the National Synchrotron Radiation Research Center in Taiwan. GD‐26 Fab/TD peptide complex crystals used 40% PEG 3350 as the protectant for data collection at cryogenic temperatures. The data set was collected to 1.45 Å resolution by using the NSRRC Taiwan Photon Source beamline TLS‐15A1. The diffraction data set was processed by using the software HKL‐3000 [52]. The GD‐26 Fab/TD peptide crystal belonged to space group *P*2_1_2_1_2_1_ with unit‐cell dimensions of *a* = 51.43 Å, *b* = 74.34 Å, and *c* = 108.1 Å.

The crystal structure of GD‐26 Fab/TD peptide was solved by molecular replacement with the software *PHENIX* [53] using the structure of PDB ID: 4ZXB [54] as a search model. One Fab molecule was located. The program *Coot* [55] was used for model building. The peptide antigen model was built according to the GTGATPADD sequence, guided by a 2Fo‐Fc map. The structure was refined using *REFMAC5* [56], and the refinement gave *R*_work_ and *R*_free_ values were 0.13 and 0.178, respectively. All figures of protein structures were produced by using the *UCSF Chimera* software [57].

### Western blotting

Whole‐cell extracts were dissolved in NuPAGE LDS sample buffer (Thermo Fisher Scientific). 20, 10, 1, 0.5 and 0.1 µL of the extracts were loaded and fractionated on NuPAGE 12% Bis‐Tris gels (Thermo Fisher Scientific) under reducing conditions. Following SDS/PAGE, cell extracts were transferred onto poly(vinylidene difluoride) membranes (Merck Millipore, Burlington, MA, USA). The membranes were blocked with 5% (w/v) nonfat dry milk in the TBST buffer (50 mm Tris, 150 NaCl, 1% Tween 20, pH 7.6) for 1 h at room temperature. Following blocking, the membranes were incubated with GD‐26 IgG (1 mg·mL^−1^; 1 : 2000) or anti‐histidine tag antibody (clone HIS.H8; Merck Millipore; 1 : 2000) for 1 h at room temperature. After washing three times with the TBST buffer for 5 min, the membranes were incubated with a 1 : 100 000 dilution of anti‐mouse IgG (whole molecule)‐peroxidase antibody (Sigma‐Aldrich, St. Louis, MO, USA) for 1 h at room temperature. The membranes were washed three times with the TBST buffer, developed with the chemiluminescent horseradish peroxidase substrate (Merck Millipore) and imaged by iBright Imaging Systems (Thermo Fisher Scientific). The same protocol was applied to western blotting on purified eGFP (5–20 ng).

### ELISA

Clear flat‐bottom Maxisorp Nunc‐Immuno plates (Thermo Fisher Scientific) were coated with 100 µL of TD‐tagged eGFP ranging from 0.1 µg to 1 pg in the coating buffer (0.1 m Na_2_HPO_4_, pH 9.6) overnight at 4 °C. Following five washes with the PBST buffer (137 mm NaCl, 2.7 mm KCl, 9.7 mm Na_2_HPO_4_, 2.1 mm KH_2_PO_4_, 0.1% Tween 20, pH 7.4), the plates were blocked with 200 µL of 5% (w/v) nonfat dry milk in the PBST buffer for 1 h at room temperature. After blocking, the plates were washed five times with the PBST buffer and then incubated with 100 µL of GD‐26 IgG (1 mg·mL^−1^) with a 1 : 10 000 dilution for 1 h at room temperature. After washing five times with the PBST buffer, the plates were incubated with 100 µL of anti‐mouse IgG (whole molecule)–peroxidase antibody (1 : 100 000; Sigma‐Aldrich) for 1 h at room temperature. The plates were washed five times with the PBST buffer, developed using 100 µL of TMB substrate solution and stopped by 100 µL of 0.5 N HCl solution. Absorbance was read at 450 nm using the Powerwave XS2 Plate Reader (BioTek, Winooski, VT, USA).

### Cell culture, transfection and immunofluorescence

The eGFP gene encoding residues 1–231 with eight histidines at the N terminus and the TD peptide with a linker consisting of two glycine residues at the C terminus were optimised for expression in mammalian cells, synthesised and subcloned into the pcDNA3 plasmid digested with *Kpn*I and *Xba*I restriction enzymes (BIOTOOLS). HEK293 cells were cultured in Dulbecco's modified Eagle's medium (high glucose, pyruvate; Thermo Fisher Scientific) supplemented with 10% FBS (Thermo Fisher Scientific) at 37 °C with 5% CO_2_. HEK293 cells were seeded onto poly‐d‐lysine (Thermo Fisher Scientific)‐coated coverslips in a 12‐well plate at a density of 2.5 × 10^5^ cells per well. Two days after incubation at 37 °C with 5% CO_2_, HEK293 cells in each well were transiently transfected with 1.6 µg of plasmid DNA with the use of 1.6 µL of Lipofectamine 2000 transfection reagent (Thermo Fisher Scientific) following the manufacturer's instructions. Two days after transfection, cells were rinsed in phosphate‐buffered saline (PBS; 3 × 5 min) and fixed for 10 min in 4% (v/v) paraformaldehyde in PBS. Fixed cells were rinsed in PBS (3 × 5 min) and then permeabilised for 20 min in 0.2% (v/v) Triton X‐100 in PBS. Permeabilised cells were rinsed in PBS (3 × 5 min) and blocked with 10% (v/v) goat serum (Thermo Fisher Scientific) in the PBT buffer (PBS containing 0.5% (w/v) BSA and 0.1% (v/v) Triton X‐100) at overnight at 4 °C. Following blocking, cells were incubated with GD‐26 IgG (1 mg·mL^−1^; 1 : 1000 in PBT) for 2 h at room temperature. After rinsing for 3 × 5 min in PBS, cells were incubated with Alexa Fluor 568‐conjugated goat anti‐mouse IgG (H + L) highly cross‐adsorbed secondary antibody (Thermo Fisher Scientific; 1 : 200 in PBT) for 1 h at room temperature in the dark. After removal of the excess secondary antibody by rinsing with PBS (3 × 5 min), coverslips containing immunostained cells were mounted on microscope slides with Vectashield® Vibrance™ antifade mounting media with DAPI (Vector Laboratories, Burlingame, CA, USA). Immunofluorescence images were acquired using a Leica TCS SP5 X confocal microscope (Leica Microsystems, Wetzlar, Germany) at 63X magnification and zoom 2. The excitation wavelengths for DAPI‐stained nuclei, eGFP and Alexa Fluor 568‐labelled GD‐26 IgG were 405, 488 and 561 nm, respectively. Images were processed by imagej/fiji software (National Institutes of Health, Bethesda, MD, USA) [58].

## Conflict of interest

The authors declare no conflict of interest.

## Author contributions

P‐JP and C‐CL planned the experiments. M‐FH provided the GD‐26 IgG sequence. P‐JP, M‐HC and C‐TC performed the experiments. C‐CL conducted crystal structure analysis. P‐JP, C‐CL and AH‐JW analysed the data and wrote the paper.

### Peer Review

The peer review history for this article is available at https://publons.com/publon/10.1111/febs.16184.

## Data Availability

Structural data are available in the PDB database under the accession number of 7DOH.
